# Identification of the Urogenital Distress Inventory-6 and the Incontinence Impact Questionnaire-7 cutoff scores in urinary incontinent women

**DOI:** 10.1186/s12955-021-01721-z

**Published:** 2021-03-16

**Authors:** Katarzyna Skorupska, Magdalena Emilia Grzybowska, Agnieszka Kubik-Komar, Tomasz Rechberger, Pawel Miotla

**Affiliations:** 1 Second Department of Gynecology, Medical University of Lublin, Jaczewskiego 8, 20-954 Lublin, Poland; 2 Department of Gynecology, Gynecological Oncology and Gynecological Endocrinology, Medical University of Gdańsk, Gdańsk, Poland; 3 Department of Applied Mathematics and Computer Science, University of Life Science, Lublin, Poland

**Keywords:** Quality of life, Urogenital Distress Inventory-6 (UDI-6), Incontinence Impact Questionnaire-7 (IIQ-7), Cutoff values, Urinary incontinence

## Abstract

**Purpose:**

Urogenital Distress Inventory-6 (UDI-6), Incontinence Impact Questionnaire-7 (IIQ-7) and The International Consultation on Incontinence (ICIQ-SF) Short Form are used to diagnose individuals with urinary incontinence (UI) and to assess the impact of the dysfunction on patient quality of life. While ICIQ-SF has fixed cutoff values—UDI-6 and IIQ-7 do not. We aimed to find the cutoff scores for UDI-6 and IIQ-7 in women with UI.

**Methods:**

The study involved 205 women aged between 31 and 83 years—155 with, and 50 without UI symptoms. All participants completed all three questionnaires: ICIQ-SF, UDI-6 and IIQ-7. Patients were categorized according to their ICIQ-SF scores, as symptomatic ICIQ-SF ≥ 6 (n = 134) and asymptomatic ICIQ < 6 (n = 60). The Receiver Operating Characteristics (ROC) curve was used to test how well UDI-6 allowed a discrimination between patients suffering from UI and those who do not. Area under Curve (AUC) statistic was calculated to measure the UDI-6 and IIQ-7 Total Score efficiency.

**Results:**

The cutoff values were selected. On the basis of the ROC curve analysis, the UDI-6 Total Score of 33.33 and IIQ-7 Total Score of 9.52 were determined to be the optimal cutoff for distinguishing between symptomatic and asymptomatic women (AUC = 0.94-UDI-6 and 0.91-IIQ-7).

**Conclusions:**

For UDI-6 scores more than 33.33 indicate higher distress caused by UI symptoms. Moreover, the higher impact of UI on health- related quality of life is seen in women who scored 9 or more in the IIQ-7 questionnaire, and such women felt impaired quality of life.

Trial registration number NCT04433715, 11.06.2020 “retrospectively registered”.

## Plain English summary

Urogenital Distress Inventory-6 (UDI-6), Incontinence Impact Questionnaire-7 (IIQ-7) and The International Consultation on Incontinence (ICIQ-SF) Short Form are used to diagnose individuals with urinary incontinence and to assess the impact of the dysfunction on patient quality of life. While ICIQ-SF has fixed cutoff values—UDI-6 and IIQ-7 do not. We aimed to find the cutoff scores for UDI-6 and IIQ-7 in women with urinary incontinence in order to find individuals with symptomatic urinary incontinence. Our study involved 205 women aged between 31 and 83 years—155 with, and 50 without urinary incontinence symptoms. All participants completed all three questionnaires: ICIQ-SF, UDI-6 and IIQ-7. Patients were categorized according to their ICIQ-SF scores, as symptomatic ICIQ-SF ≥ 6 (n = 134) and asymptomatic ICIQ < 6 (n = 60). Then we tested how well UDI-6 allowed a discrimination between patients suffering from urinary incontinence and those who do not. The cutoff values were selected. The UDI-6 Total Score of 33.33 and IIQ-7 Total Score of 9.52 were determined to be the optimal cutoff for distinguishing between symptomatic and asymptomatic women. For UDI-6 scores more than 33.33 indicate higher distress caused by urinary incontinence symptoms. Moreover, the higher impact of urinary incontinence on health- related quality of life is seen in women who scored 9 or more in the IIQ-7 questionnaire, and such women felt impaired quality of life.

## Introduction

The incidence of urinary incontinence (UI) in the general population of women differs depending on the methodology of the study. Overall, from 13 to 39% of all women aged between 15 and 87 years report one or more of the UI types [1]. The evaluation of pelvic floor treatments has changed significantly in recent years. Initially focused on the assessment of symptoms, Quality of Life (QoL) or patient satisfaction, it has gradually turned to a new concept, such as Patient Reported Outcome (PRO) [2]. Questionnaires are used as PROs in order to identify and diagnose individuals with dysfunction, assess the severity of this dysfunction, examine the impact of dysfunction on patient QoL and measure improvement and/or satisfaction after treatment. There are many different procedures available to treat UI and a good outcome measure is necessary to prove benefits of particular methods. Various measures are available to assess the impact of UI on Health Related Quality of Life (HRQoL). Urogenital Distress Inventory-6 (UDI-6) and Incontinence Impact Questionnaire-7 (IIQ-7) are urinary incontinence-specific psychometric questionnaires and are employed in conjunction with one another [3]. The International Consultation on Incontinence Modular Questionnaire (ICIQ-SF) Short Form is considered “the gold standard” in the detection of UI [4]. While ICIQ-SF has fixed cutoff values [5]—UDI-6 and IIQ-7, which are commonly used in the assessment of UI, do not have these. Therefore, the aim of the study was to find the cutoff scores for UDI-6 and IIQ-7 in women with UI.

## Materials and methods

**Fig. 1:**
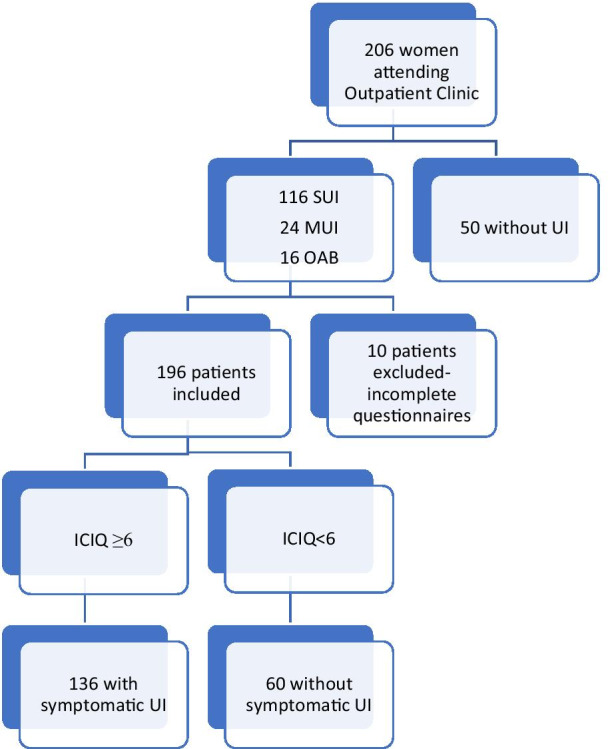
Study flow chart

The study protocol was conducted in accordance with the European Communities Council Directive of 22 September 2010 (2010/63/EU) and Polish legislation acts and was approved by the Local Ethics Committee. Before inclusion, all patients gave written informed consent for the participation in the study. The study involved a total of 205 women aged between 31 and 83 years who were recruited from women attending the Gynecological Outpatient Clinic due to UI symptoms. The patient’s medical history was taken and urogynecological examination was performed according to the International Continence Society (ICS) standards [6, 7]. During examination, the assessment of the degree of Pelvic Organ Prolapse (POP) was based on the POP-Q scale [8]. Symptomatic POP was an exclusion criterium, as was lower urinary tract symptoms (LUTS) resulting from urinary tract infections, stones, tumors, neurological diseases and urogenital atrophy. Urodynamic investigation, including uroflowmetry, cystometry and pressure flow studies (Medtronic, duet Logic G/2), were performed in women reporting LUTS symptoms, and enabled us to diagnose the type of UI according to Drake. Based on physical examination and urodynamics (which objectively confirm the diagnosis of UI), 50 patients did not present any UI type [7]. All participants were asked to complete polish version of 3 questionnaires: ICIQ-SF, UDI-6 and IIQ-7 to assess the subjective impact of UI on HRQoL. We identified ICIQ-SF score of < 6 as a marker of asymptomatic UI patient. Based on a study by Karmakar et al. [5], a postoperative ICIQ-SF score of < 6 was likely to translate to a patient-reported successful postoperative outcome according to the Patient Global Impression of Improvement (PGI-I) [5]. Therefore, patients were categorized according to their ICIQ-SF scores, as symptomatic ICIQ-SF ≥ 6 (group 1) and asymptomatic ICIQ < 6 (group 2) [5]. The enrolment process is shown in Fig. 1.

### Questionnaires

The ICIQ-SF is a questionnaire that evaluates the frequency, severity and the impact of UI on QoL in research and clinical practice. It was first introduced in 1999. ICIQ-SF’s level of validation according to the International Consultation on Incontinence (ICI) grades is A, meaning that it is a highly recommended questionnaire. Moreover, its validity, reliability and responsiveness to change has been confirmed [9]. The questionnaire consists of four items: Frequency of UI, Amount of leakage, Overall impact of UI and Self-diagnostic item. ICIQ-SF questions use 5-point Likert scales to assess the presence or absence of symptoms and their severity. The overall score range is from 0 to 21, with greater values indicating increased symptom severity. The cutoff score for differentiating women with and without UI is 6 [5]. ICIQ-SF has been validated as approximately 90% sensitive and as an 85% specific tool for success or failure assessment in a group of post-operative women [5].

UDI-6 is a short version of a condition-specific quality of life instrument—UDI, and was introduced in 1994 [3]. Presently, due to its feasibility, UDI-6 is much more often used than its longer version. UDI-6 level of validation according to ICI grades is A [9]. UDI-6 consists of 6 items: 1—Frequent urination, 2—Leakage related to feeling of urgency, 3—Leakage related to activity, 4—Coughing, or sneezing small amounts of leakage (drops), 5—Difficulty emptying the bladder, and 6—Pain or discomfort in the lower abdominal or genital area. Higher scores in UDI-6 indicate higher disability. Total score is from 0 to 100 [10].

IIQ-7 is a urinary incontinence-specific psychometric questionnaire. This questionnaire assesses the psychosocial impact of UI in women. The IIQ-7 level of validation according to ICI grades is A [9]. It consists of 7 items: 1—Household chores, 2—Physical recreation, 3—Entertainment activities, 4—Travel > 30 min away from home, 5—Social activities, 6—Emotional health (nervousness, depression, etc.), 7—Feeling frustrated; which is subdivided into 4 domains: PA—physical activity (items 1 and 2), TR—travel (items 3 and 4), SA—social activities (item 5), and EH—emotional health (items 6 and 7). Total score ranges from 0 to 100 [11].

UDI-6, IIQ-7 and ICIQ have been successfully translated and validated into Polish [12].

#### Statistical analysis

Statistical analysis was performed with R open source software [13], and most of the calculations and visualizations were made using functions from the Optimal Cut points package [14]. The results between UDI-6 and ICIQ-SF, as well as, IIQ-7 and ICIQ-SF were compared using Pearson correlation coefficient to assess the criterion validity. All continuous variables were expressed as means and standard deviations. A two-sided t-test was applied for the comparison of the means between symptomatic and asymptomatic groups, at the significance level α = 0.05. A Receiver Operating Characteristics (ROC) curve was used to test how well UDI-6 allowed the discrimination between patients suffering from UI and those who not presenting any symptoms. It was also used to verify how well IIQ-7 distinguishes patients with QOL who are also affected by UI. In addition, the AUC (Area under Curve) statistic was calculated to measure the UDI-6 and IIQ-7 Total Score efficiency in the aforementioned prediction. A ROC curve is a graph illustrating the diagnostic ability of a binary classifier system when its discrimination threshold is varied. ROC is created when the true-positive rate (sensitivity) is plotted against the false-positive rate (1-specificity). It is then used to generate the optimal cutoff level for correctly identifying diseased or non-diseased subjects. In our study, the Youden index (J) was indicated for this purpose, which is the farthest point on the ROC curve from line of equality (diagonal one). This means that J maximizes the difference between sensitivity and 1-specificity [15]. The verification of the classification was performed on the base of Cohen's kappa (κ), which represents the chance-corrected proportional agreement [16]. According to the rule of thumb used in establishing sample size, an ideal ratio of respondents to items is 10:1, which means that at least 10 participants per group is recommended for each scale item for psychometric work, so a sample size of at least 70 UI women was required [17].

## Results

We ascertained that 154 (75.1%) patients out of 205 had UI. Herein, the mean age was 57.6 ± 10.8 years and mean body mass index (BMI) was 27.5 ± 4.6 kg/m^2^. Based on urodynamics findings, stress urinary incontinence (SUI) was diagnosed in 115, overactive bladder (OAB) in 16 and mix urinary incontinence (MUI) in 24 patients. OAB diagnosis was based on the finding of detrusor overactivity in urodynamics. Fifty patients did not report any UI symptoms. Ten patients were excluded from the study due to incomplete answers to the questionnaires. After dichotomizing patients according to their ICIQ-SF results, we separated out two groups: 134 patients (69.1%) with ≥ 6 ICIQ—symptomatic UI and 60 patients (30.9%) < 6 ICIQ—asymptomatic. Women from both groups did not complain of any POP and did not undergo any anti-incontinence surgery in the past. Mean ICIQ-SF Total Score and Total UDI-6 and IIQ-7 scores for group 1 (ICIQ ≥ 6) and group 2 (ICIQ < 6) women are presented in Table 1.

**Table 1:** Mean results of The International Consultation on Incontinence Modular Questionnaire (ICIQ-SF) Short Form, Urogenital Distress Inventory-6 (UDI-6) and Incontinence Impact Questionnaire-7 (IIQ-7)

Questionnaire	Group 1 (ICIQ ≥ 6) Mean ± SD	Group 2 (ICIQ < 6) Mean ± SD	p value
ICIQ	13.5522 ± 4.20	0.7333 ± 1.64	< 0.001
UDI-6	62.0564 ± 19.30	11.0185 ± 19.58	< 0.001
IIQ-7	56.9119 ± 26.19	8.2539 ± 21.47	< 0.001

In the study, the Pearson's correlation coefficient between UDI-6 and ICIQ-SF scores was 0.81, and between IIQ-7—ICIQ-SF scores—0.79. This indicates high positive linear correlations between the referral and the analyzed questionnaires.

**Fig. 2:**
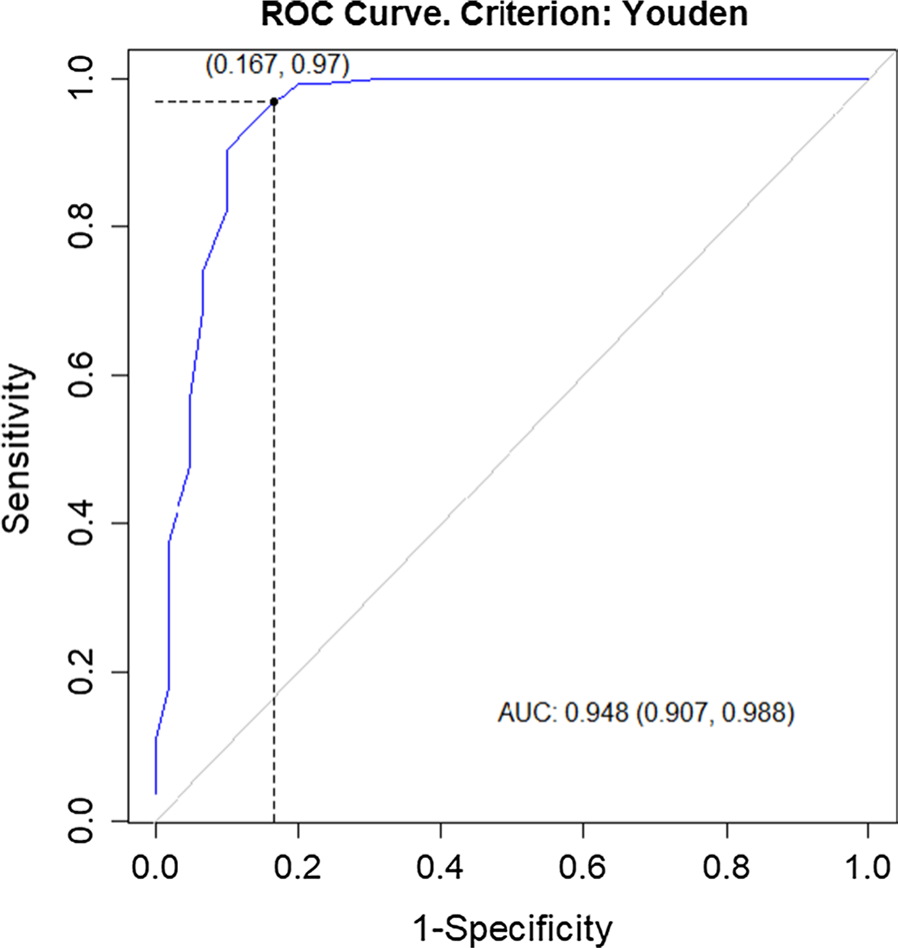
ROC (receiver operating characteristics) curve for Urinary Incontinence for Urinary Distress Inventory (UDI-6)

**Fig. 3:**
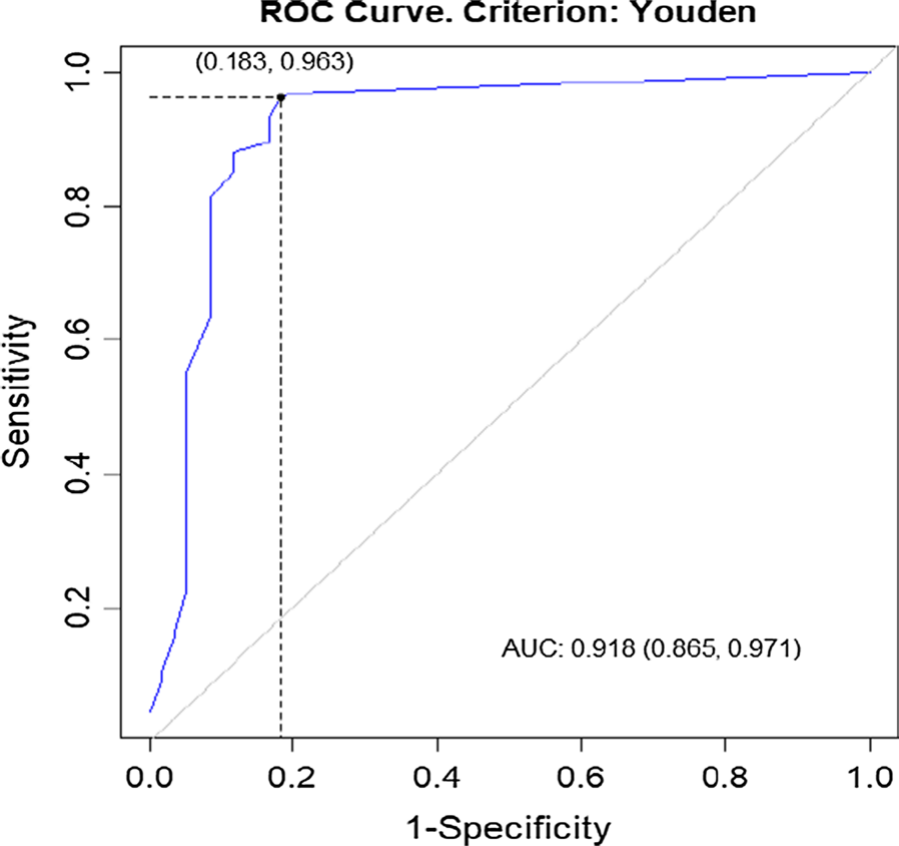
ROC (receiver operating characteristics) curve for Urinary Incontinence for Incontinence Impact Questionnaire (IIQ-7)

On the basis of the ROC curve analysis, the UDI-6 Total Score of 33.33 and IIQ-7 Total Score of 9.52 were determined to be the optimal cutoff for distinguishing between symptomatic and asymptomatic women (Figs. 2 and 3). The hypothesis that patients who scored in UDI-6 and IIQ-7 more than the cutoff values suffer from UI resulted in the true-positive rate of 97% (for UDI-6) and 96% (for IIQ-7) (sensitivity), and represented the proportion of symptomatic patients who were properly classified by the UDI-6 and IIQ-7 Total Score as “symptomatic group”. The false-positive rate of 16.7% (for UDI-6) and 18.3% (for IIQ-7) (1-specificity) represented the ratio of patients with urinary tract dysfunction who were improperly classified as “symptomatic group” (Fig. 2).

The area under the ROC curve (AUC) for UDI-6 and IIQ-7 was equal to 0.94 and 0.91 relevance. These values indicate high effectiveness of classifying the subjects into “symptomatic” and “asymptomatic” groups for both diagnostic tests [18].

Cohen’s kappa calculated on the basis of misclassification matrices was applied for the verification of the classification model. The values, 0.8263 for UDI-6 and 0.8014 for IIQ-7, indicate high agreement between classification by ICIQ-SF and by the studied diagnostic tests mentioned above.

## Discussion

Urinary incontinence is a huge heterogeneous problem worldwide. To assess the impact of UI comprehensively, it is necessary to measure both the level of an individual’s symptoms and the extent to which they impair their life. The use of questionnaires to assess outcomes in urogynecology has been increasing. UDI-6 and IIQ-7 are well known broadly used condition- specific questionnaires which were previously validated into Polish [12] The ICIQ-SF is a condition- specific questionnaire. It has established cutoff scores [5], therefore, it was selected as the comparative tool to determine the UDI-6 and IIQ-7 cutoff values. ICIQ-SF cutoff scores were established in a study where patients underwent UI-intervention, and these cutoff scores predicted a successful PRO according to the PGI-I questionnaire. Hence, the Authors claim that postoperative ICIQ-SF < 6 is associated with a patient-reported successful outcome on the PGI-I. In addition, ICIQ-SF has severity categories established by Klovning et al. as slight (1–5), moderate (6–12), severe (13–18) and very severe (19–21) that were assessed in a group of 343 UI women [19]. Due to the fact that UDI-6 is a condition-specific questionnaire, it has previously been used in studies where authors applied it as a tool in distinguishing symptomatic and asymptomatic UI patients. Among others, Gafni-Kane et al. created predictive modeling and threshold scores for women seeking care due to UI. They discovered that a UDI-6 score of 25.00 (83.33% sensitivity and 83.59% specificity) had excellent discriminatory accuracy in distinguishing care and non-care seekers [20]. Beyond the aforementioned, Cocci et al., based on an analysis of baseline characteristics, determined that a cutoff value of 9.0 on the UDI-6 predicted postoperative SUI with 62% specificity, 72% sensitivity and 66% accuracy. According to their results, in multivariate logistic regression analysis, a preoperative UDI-6 ≥ 9.0 was an independent predictor of persistent SUI after transobturator tape procedure [21].

IIQ-7 is a short version of the IIQ questionnaire. The total IIQ score ranges from 0 to 400 [22]. In the study of Corcos et al., the authors established levels of deterioration of QoL in patients with UI according to the long version of the IIQ. Here, a score of less than 50 on the IIQ would be representative of good QoL, between 50 and 70 would be moderate QoL, and greater than 70 would be indicative of poor QoL. Corcos et al. claimed that the identification of three levels of QoL should be useful in clinical decision-making. Accordingly, women that score less than 50 should be discouraged from surgical intervention and patients with SUI and IIQ score greater than 70 will probably see a greater change after an intervention [23]. Botros et al. [24] tried to identify normative values for UDI-6 and IIQ-7 within the general female population, and found that 10% scored above 16.7 and 4.8 on the UDI-6 and IIQ-7, respectively. They used data from 181 female patients, out of whom 104 were incontinent. Botros et al. noted that ninety percent of all discovered incontinent patients scored < 44.4 in UDI-6 and < 23.8 in IIQ-7, and the highest scores in the continent population were 33.3 and 57.1, respectively.

Based on our results the optimal cutoff score for distinguishing between symptomatic and asymptomatic UI women is for UDI-6—33.33, with scores more than 33.33 indicating higher distress caused by UI symptoms. Moreover, the higher impact of UI on HRQoL is seen in women who scored 9 or more in the IIQ-7 questionnaire, and such women felt impaired quality of life. The limitation of the study is the single setting and the fact that UDI-6 and IIQ-7 cutoff scores were established based upon ICIQ-SF cutoff scores which were associated with a patient-reported successful outcome on the PGI-I following surgical treatment with a midurethral sling in women. Patients with UI were treated in our study as one group (symptomatic) without dividing into SUI, MUI and OAB subgroups—which would have given possibility for further studies. The results of this study may help clinician to find symptomatic UI patients and refer them for further diagnosis and treatment. Up till now only change in scores was an indication of improvement or deterioration after the treatment applied. The cutoff scores give the researchers the ability to refer their results to the established thresholds. Therefore allowing wider use of the patient reported outcomes. Further studies are needed to confirm those results in different populations.

## Data Availability

Not applicable.
